# The SBAR tool for communication and patient safety in gynaecology and obstetrics: a Tunisian pilot study

**DOI:** 10.1186/s12909-024-05210-x

**Published:** 2024-03-05

**Authors:** Dhekra Toumi, Wafa Dhouib, Ines Zouari, Imen Ghadhab, Mouna Gara, Olfa Zoukar

**Affiliations:** 1 University of Monastir, Monastir, Tunisia; 2 Department of Epidemiology and Preventive Medicine, University of Monastir, Monastir, Tunisia

**Keywords:** Health communication, Patient safety, Education

## Abstract

**Background:**

In healthcare, inadequate communication among providers and insufficient information transmission represent primary contributors to adverse events, particularly in medical specialties such as obstetrics and gynecology. The implementation of SBAR (Situation-Background-Assessment-Recommendation) has been proposed as a standardized communication tool to enhance patient safety. This study aims to evaluate the knowledge, attitudes, and practices related to SBAR communication through a pilot study conducted in a middle-income country.

**Methods:**

This prospective longitudinal study took place in the gynecology-obstetrics department of a Tunisian university hospital from May to June 2019. All medical and paramedical staff underwent comprehensive theoretical and practical training through a 4-hour SBAR simulation. To gauge participants’ knowledge, anonymous multiple-choice questionnaires were administered before the training initiation, with a second assessment conducted at the end of the training to measure satisfaction levels. Two months later, the evaluation utilized questionnaires validated by the French National Authority for Health (HAS).

**Results:**

Among the 62 care staff participants in this study, a majority (89%) demonstrated a low level of knowledge regarding the SBAR tool. The majority (75.8%) expressed enjoyment with the training and indicated their intention to implement changes in their practice by incorporating the SBAR tool in the future (80.7%). Notably, over half of the participants (79%) expressed satisfaction with the training objectives, and 74% reported acquiring new information. Evaluation of the practice revealed positive feedback, particularly in terms of clarity, the relevance of communication, and the time spent on the call.

**Conclusion:**

Our pilot study showed that the majority of professionals on the ward had little knowledge of the SBAR tool, a good attitude and a willingness to put it into practice. It is essential that healthcare managers and professionals from all disciplines work together to ensure that good communication practice is developed and maintained. Organisations, including universities and hospitals, need to invest in the education and training of students and health professionals to ensure good quality standardised communication.

## Background

The World Health Organization (WHO) identified communication as one of five priorities for decreasing adverse events in health care (AEHI) and so hat increasing overall patient safety [1]. Miscommunication between healthcare providers and the lack of information transmission constitute significant sources of diagnostic errors and adverse events in health care across various medical specialties [2–5]. Hospitals in low- and middle-income countries experience a staggering 134 million adverse events, leading to 2.6 million deaths and contributing to approximately two-thirds of the global burden, which includes the loss of disability-adjusted life years [6, 7]. The deficiency in communication can be attributed to the heterogeneity arising from factors such as multi-professionality, personality variations, and cultural and behavioral differences among healthcare providers. Consequently, standardizing communication between providers becomes essential to mitigate these challenges and enhance overall communication effectiveness [8]. The SBAR (Situation-Background-Assessment-Recommendation) tool serves as an interprofessional communication technique, offering a systematic approach to enhance quality and safety by addressing barriers stemming from factors such as multi-professionality, personality differences, and cultural variations among healthcare providers. Notably, this technique has gained widespread acceptance and is now integrated into the standard of care in numerous countries, including the USA, Canada, UK, Australia, Spain, France, and others [8]. In France, the High Authority for Health has introduced a French adaptation of the SBAR tool known as SAED. This adaptation aims to prevent AEHI that may arise from communication misunderstandings among professionals. The implementation of SBAR is designed to facilitate clear and concise documented communication, ultimately reducing the likelihood of oversights in healthcare processes [9]. Due to the increased susceptibility of obstetric and gynecological care providers to medical malpractice [10], the International Federation of Gynecology and Obstetrics (FIGO) has incorporated the use of this new communication tool into certain Clinical Protocols [11]. Tunisia, as a developing country, contends with a health system marked by various difficulties and challenges [12]. In Tunisia, the incidence of AEHI stands at approximately 10%, with 60% of these incidents deemed avoidable. Alarmingly, 21% of these events lead to patient fatalities. To address this issue, the Tunisian National Evaluation and Accreditation Body in the Health Sector (called “INEAS”), under the Services Competitiveness Support Programme (called “PACS”), has actively promoted the adoption of the SBAR tool. This initiative is aimed at securing oral and telephone communications among healthcare providers and has been explicitly mentioned in the accreditation manual for second and third-line health establishments, focusing on enhancing intra- and inter-team communication [13]. Several articles have discussed the impact of the SBAR tool on patient safety [14].

Nevertheless, up to the present moment, the assessment and standardization of interpersonal communication are underappreciated and underutilized within the Tunisian health sector. Additionally, there is a lack of Tunisian studies demonstrating the utilization of the SBAR communication tool in healthcare institutions. In an effort to prevent AEHIs, the Obstetrics and Gynecology Department initiated a pilot study, implementing training on the SAED tool to promote clear and concise communication among healthcare professionals. The study’s objectives were to evaluate the knowledge and attitudes towards the SBAR communication tool prior to training and to assess satisfaction and practice regarding the tool after the training.

## Methods

### Study design

This was a descriptive longitudinal prospective study conducted in the gynecology-obstetrics department of a Tunisian university hospital, spanning the period from May to June 2019.

### Setting

The Tunisian university hospital, encompassing all medical and surgical specialties, includes the gynecology department. The hospital employs twelve specialist doctors, 15 nurses, 14 midwives, 30 trainees, and 10 support staff. The medical team consistently conducts continuing medical education, incorporating pre- and post-training assessments for all healthcare workers (HCWs) within the department. In May 2019, SBAR training was facilitated by four doctors who received specialized training on this tool. Subsequently, participants were monitored for a duration of two months.

#### SBAR Training

The training was structured into three main components. In Part 1, participants underwent a 60-minute, 40-slide PowerPoint presentation introducing the significance of communication in healthcare. The pedagogical objective was to cultivate an understanding of the crucial role effective communication plays in healthcare, along with familiarizing participants with the SBAR communication tool.

Transitioning to Part 2, a simulation of information transmission was executed using the SBAR tool. This phase aimed to provide participants with hands-on experience in the practical application of the SBAR tool through pre-established scenarios, utilizing blank SBAR materials. It commenced with an initial role-playing session led by investigators, followed by a second session with active participant involvement. Throughout this phase, investigators offered corrections and feedback on communication aspects requiring improvement. The training culminated in a third role-playing session facilitated by investigators to reinforce key concepts and ensure a comprehensive understanding among participants.

In the final segment, Part 3, the training session concluded with a summary emphasizing the benefits of the SBAR tool. The objective here was to underscore the positive impact of the SBAR tool on enhancing communication among healthcare workers.

### Participants

#### Inclusion criteria

All the medical and paramedical staff of the gynecology-obstetrics department who participated in a training session; which took place at the beginning of May 2019 within the service, “Communication between Health Professionals: Oral Communication Protocol”.

#### Non inclusion criteria

Healthcare staff who were on leave or did not agree to take part in the study.

### Variables and data collection

Before the commencement of the study, the investigators provided a detailed explanation of the study’s various stages, emphasizing that participation was entirely voluntary.

During the training session, a slideshow was presented to underscore the significance of communication among health professionals and to introduce the SBAR tool. This was followed by a role-playing exercise conducted in pairs.All participating staff members were assessed before, immediately after, and two months after the training:

A preliminary anonymous multiple-choice questionnaire was administered before the training to evaluate the knowledge of the participating professionals.

Following the training, a second anonymous questionnaire was completed by the participants to assess their satisfaction and the relevance of the training topic. Participants were asked to rate their satisfaction using a Likert scale, with scores ranging from 1 (“lowest satisfaction”) to 9 (“highest satisfaction”).

The numerical variable was subdivided into 3 items:

1–3 corresponds to “Not Satisfied.

4–6 corresponds to “Moderately Satisfied.

7–9 corresponds to “Very Satisfied.

After a span of two months and during the final week of the internship, two additional questionnaires were distributed to the staff, evaluating phone calls using the SBAR tool. One questionnaire was designed for the calling professional, and the other for the called professional. It’s important to note that a healthcare worker could respond to both questionnaires if they were involved in both situations (as the caller and the called party). The completed questionnaires were then collected with the assistance of the department secretary.

**All four questionnaires were based on the French adaptation of the Anglo-Saxon SBAR tool developed by the French National Authority for Health (HAS)** [9].

### Statistical analysis

The results were documented and analyzed using Excel. Percentages were computed for qualitative variables, while means, standard deviations, and the range of extreme values were calculated for quantitative variables.

## Results

**Fig. 1:**
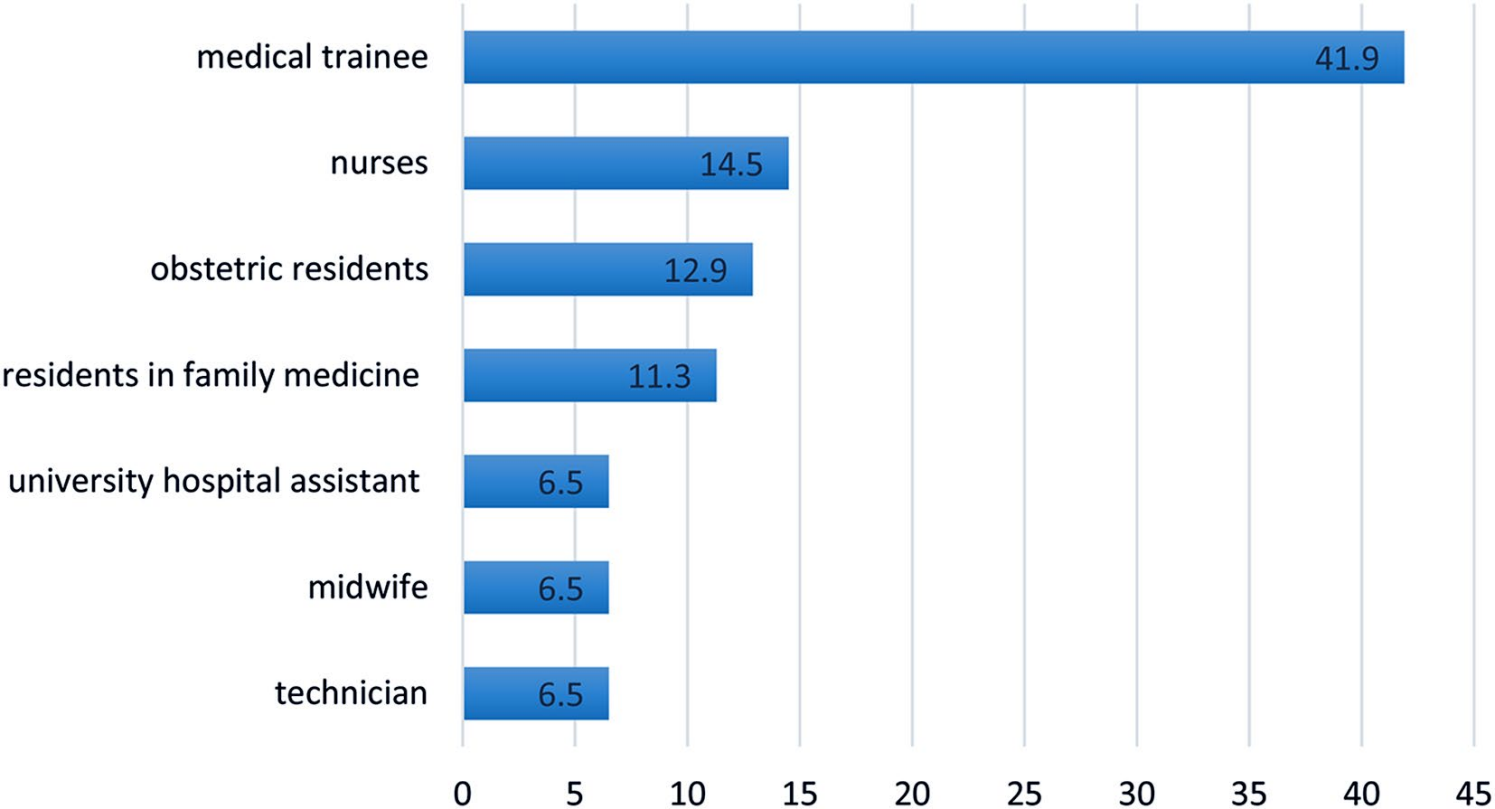
Percentage breakdown of training participants by professional group

Out of the entire staff in the gynaecology-obstetrics department, 62 healthcare personnel participated in the training. The average age of the participants was 27 ± 4 years, with age extremes ranging from 23 to 48 years. The sex ratio was 0.34 M/F. Medical trainee constituted the majority at 41.9%, followed by paramedics at 27.5%, including 14.5% represented by nurses (Fig. 1).

### Assessment of knowledge and practice of the SBAR tool

**Fig. 2:**
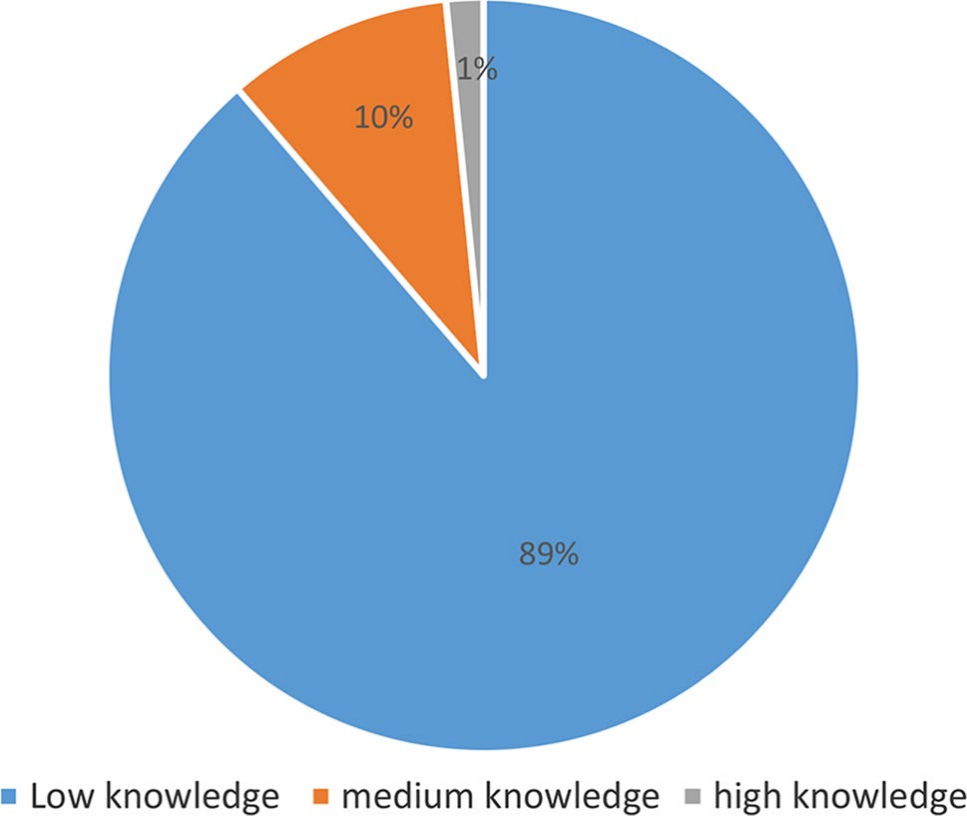
Distribution of participants according to their knowledge of SBAR

Concerning knowledge and practice before the training, it was observed that 89% of the participants had low knowledge of the SBAR tool, with only 1% possessing high knowledge of the tool. Additionally, 10% reported using the tool frequently, while 74.2% had never used it (Fig. 2).

### Evaluation of the training

**Fig. 3:**
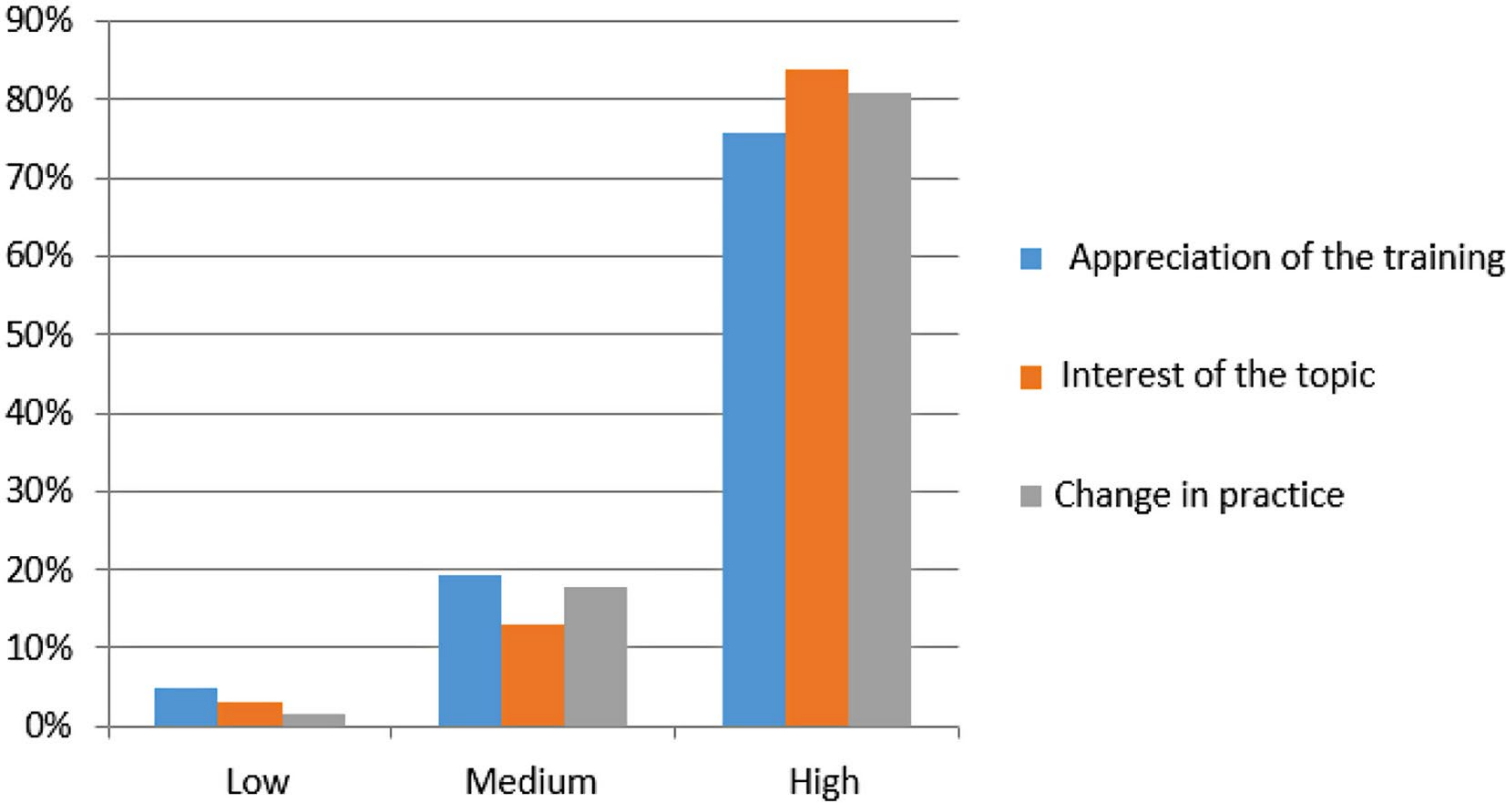
Distribution of participants according to their appreciation of the training

The majority of participants expressed positive sentiments towards the training, with 75.8% reporting enjoyment. Additionally, a significant percentage indicated interest in the training topic (83.9%), and the majority planned to implement changes in their practice by incorporating the SBAR tool in the future (80.7%) (Fig. 3).

**Fig. 4:**
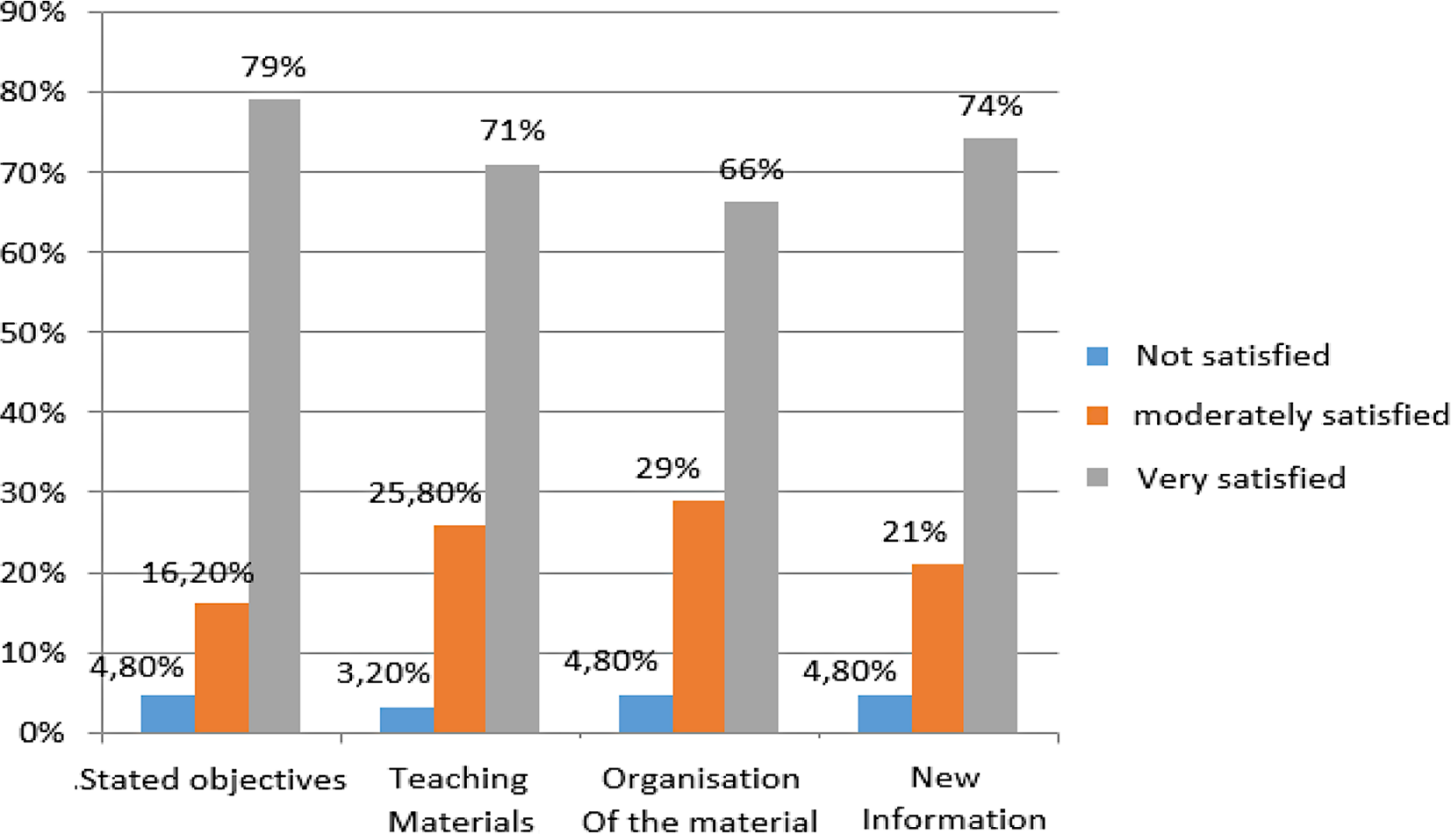
Distribution of participants according to their satisfaction with the training

Regarding satisfaction, a substantial percentage of participants expressed contentment with various aspects of the training. Specifically, 79% reported satisfaction with the training objectives, 71% with the provided teaching material, and 66% with the organization of the material. Furthermore, the majority of participants (74%) indicated that they had acquired new information through the training (Fig. 4).

**Fig. 5:**
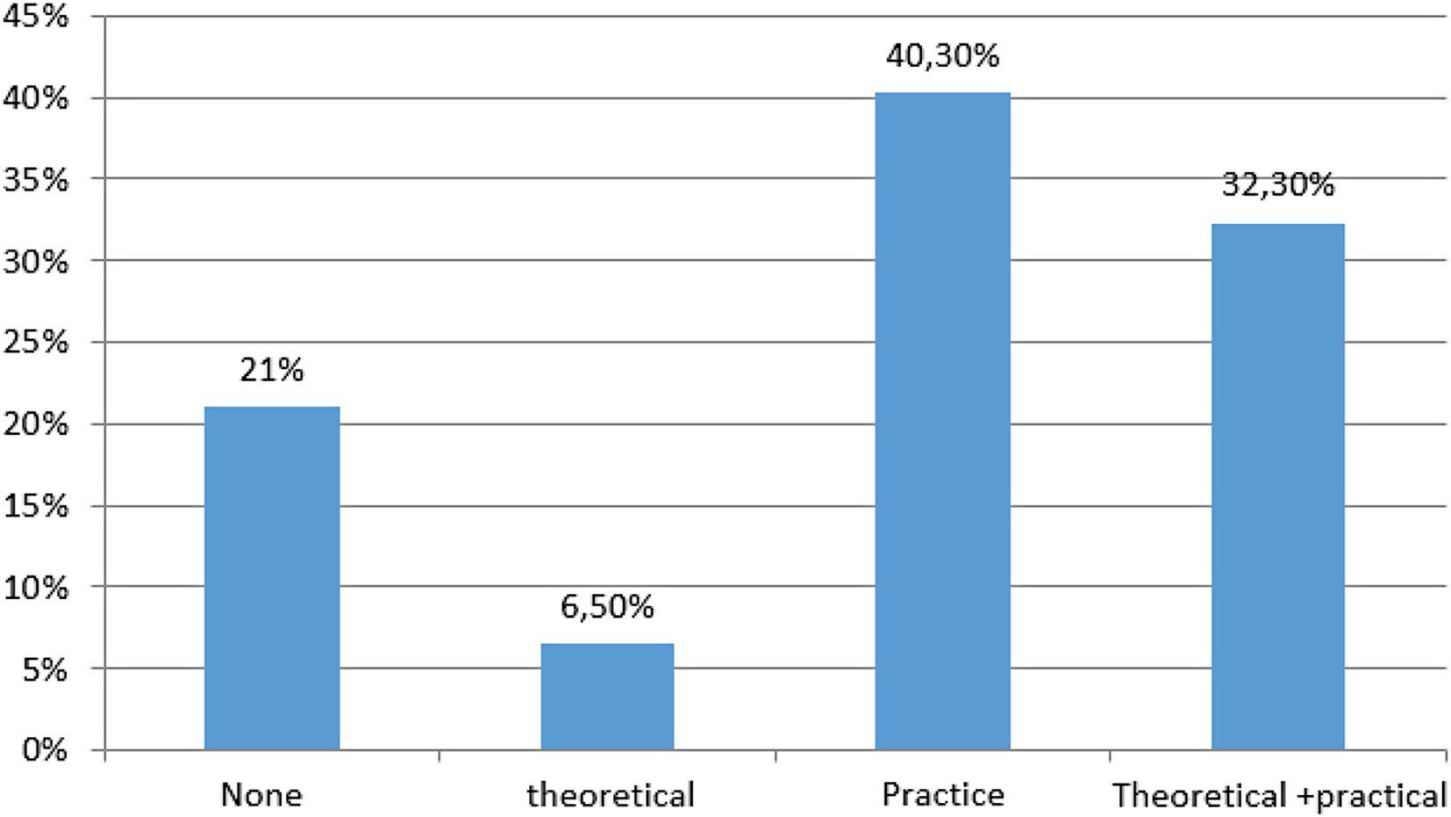
Distribution of participants’ opinions according to their practical or theoretical needs after the course

Following the training, 79% of the participants expressed a desire for further training. Among them, 32.3% indicated a need for both theoretical and practical training, while 40.3% specified a requirement for practical training alone (Fig. 5).

### Practice assessment

**Among all participating professionals, 32 individuals responded to the SBAR tool evaluation questionnaire for phone calling, yielding an overall response rate of 51.6%. Among them, 32 were callers, and 30 were respondents on the phone call.**

#### Assessment the caller

In the department or in obstetric and gynecological emergencies, the majority of the **32** calling professionals (72.2%) reported that the preparation time for the phone call was deemed satisfactory and appropriate. Additionally, 38.9% did not encounter any difficulties during the phone call. As for the response from the called professional, 72.2% found it to be very satisfactory, while 11.1% felt that the response to the call was not satisfactory.

#### Assessment of the appellant

In the department or in obstetric and gynecological emergencies, the clarity of the formulation of data was predominantly rated as excellent for all items by the majority of participants. Indeed, **72.2% of the thirty called participants**, found the assessment of the situation and the request formulated to be very clear. On the other hand, 38.9% of the participating professionals assessed the clarity of the context of the situation cited as average and 8.3% even found it weak.

**Fig. 6:**
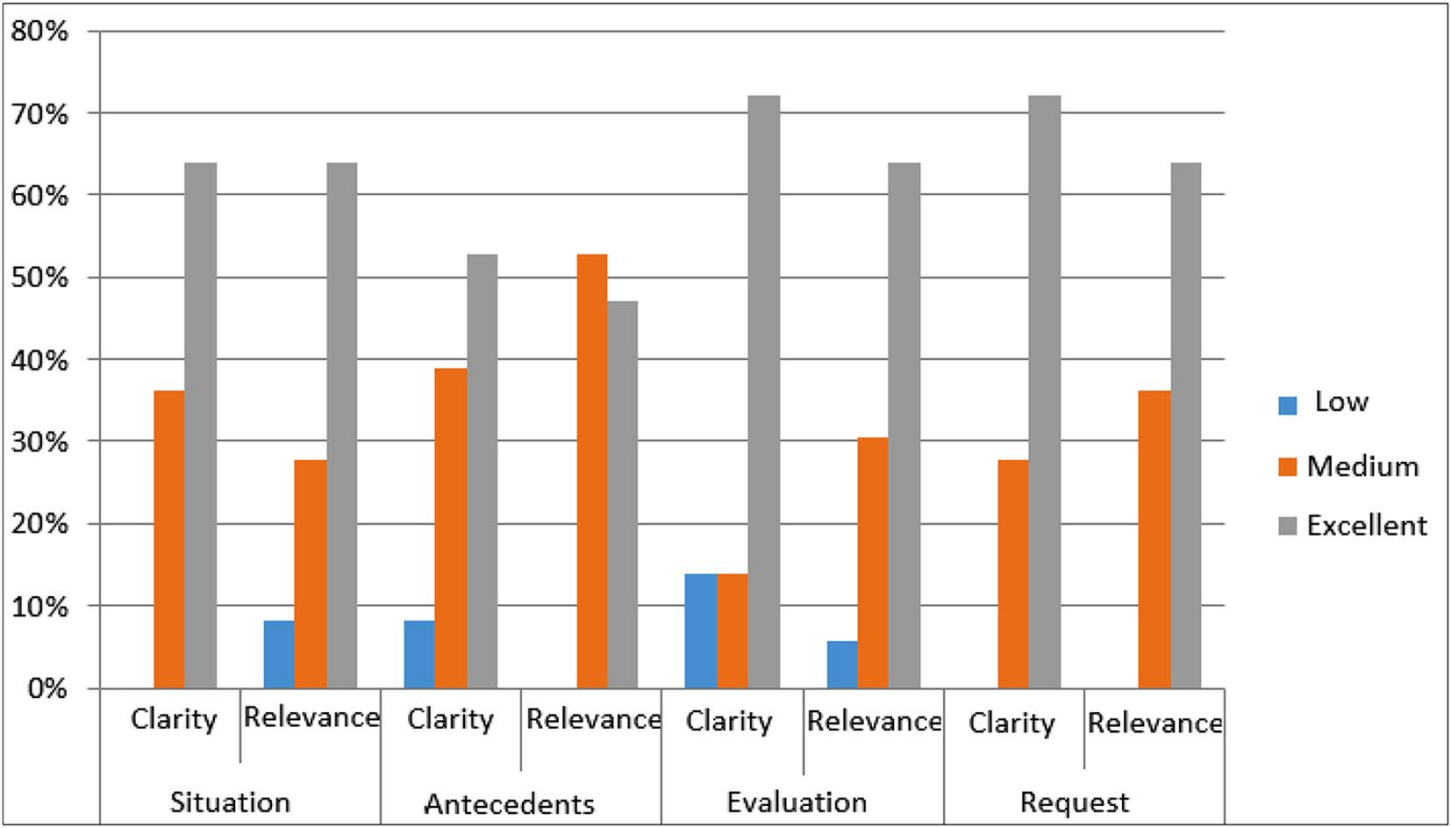
Distribution of participants according to the evaluation of the phone call

On the other hand, more than half (52.8%) of the participants called rated the relevance of the data provided regarding the context of the situation as average. On the other hand, 63.9% of the callers considered the information exchanged regarding the situation, its evaluation and application to be of excellent relevance (Fig. 6).

Participants rated the call time as follows: 78% considered it excellent, 14% rated it as average, and 8% perceived it as poor.

## Discussion

This pilot study assessed the implementation of the SBAR tool in an obstetrics and gynecology department within a middle-income country. The collaboration involved the committee of continuous professional development of paramedics with the aim of enhancing oral communication within care teams. The findings revealed a low awareness of the SBAR tool among the majority of professionals in the service. This is noteworthy, considering that the SBAR tool has been recommended by the WHO for several years and has become a standard of care in many countries [8]. The majority of the professionals were satisfied with our training and intended to use this tool in the future. The practice was mostly well appreciated by evaluating it on the clarity, the relevance of the communication and also on the time spent on the call. This initiative is the first to be undertaken in Tunisia and was developed among other projects; as part of the accreditation of the University Hospital being the first Tunisian public health institution visited for accreditation for the Support to the Competitiveness of Services (PACS) project.

The SBAR tool has been previously employed at St. Joseph Medical Center in Bloomington, Illinois, United States, during the period from 2002 to 2005. An interdisciplinary team, comprising members from nursing, pharmacy, and medical imaging, convened regularly over a year to devise a plan for the widespread implementation and dissemination of the SBAR tool across all departments. Protocols and recommendations for usage were formulated during these meetings. Simultaneously, staff underwent training in the tool’s utilization, and their practices were observed and analyzed by the project’s pilot team. The audit results were overall satisfactory, with the rate of SBAR utilization by multidisciplinary teams reaching close to 96%. This led to improved communication speed and accuracy, consequently reducing the number of undesirable events [15].

It is noteworthy that a study conducted in 2009 demonstrated the significant impact of the SBAR tool on various healthcare outcomes. The findings revealed a remarkable reduction in in-hospital mortality by 11%, a 65% decrease in adverse events, an 8% reduction in cardiac arrests, and an impressive 83% reduction in methicillin-resistant Staphylococcus aureus (MRSA) bacteraemia. Additionally, the time taken to transfer patients was substantially reduced from approximately 45 min to just seven minutes. These results underscore the effectiveness of the SBAR tool in improving patient safety and healthcare outcomes [16].

In our discussion, we contextualize our study within the broader landscape delineated in the systematic review of team interventions. This approach allows us to draw connections between our findings and the existing body of knowledge on interventions aimed at enhancing teamwork within healthcare settings [17].

Our study, focusing on the implementation of the SBAR tool in an obstetrics and gynecology department within a middle-income country, falls under the category of “Tools” for structuring teamwork. The SBAR tool, designed to enhance oral communication, aligns with tools mentioned in the literature that facilitate communication.

A notable trial published in 2012 explored the large-scale, long-term (2 years) implementation of SBAR in a network of hospitals. The article provides detailed insights into the deployment steps, showcasing a highly structured and mandatory training program for all staff. The training involved dedicated sessions and recurring role-playing exercises. After two years, 73% of nurses reported using SBAR correctly and regularly. Barriers to SBAR utilization included the reluctance of some doctors, existing habits and effective communication practices, and a lack of SBAR adoption by other colleagues in the department. This study serves as a valuable reference for understanding the challenges and successes associated with long-term SBAR implementation [18].

In France, the development of the SBAR tool has been incorporated into the framework of the national program for patient safety from 2013 to 2017 (PNSP). Many French health establishments have proactively chosen to implement communication tools, including SBAR, with the aim of enhancing and securing the patient’s journey through the healthcare system. This aligns with a broader national effort to prioritize patient safety and improve the overall quality of healthcare delivery [9].

In Belgium, particularly at the Brugmann University Hospital, the SBAR project implementation commenced in November 2016. The initiative involved an extensive training program, spanning 50 to 60 half-days spread over 5 months. A total of 1230 employees from the nursing and paramedical department, as well as geriatric and rehabilitation doctors, underwent training in the SBAR method.

Furthermore, Australian authors developed a variation of SBAR called ISBAR (Identify, Situation, Background, Assessment, Recommendation), which was introduced to final year medical students. The students who received ISBAR training demonstrated improved communication, conveying more information clearly during simulation sessions compared to students who had not undergone ISBAR training. This highlights the efficacy of structured communication tools in enhancing communication skills among healthcare professionals [19]. Several studies have targeted medical, pharmacy, nursing and care assistant students [20–23].

The implementation of the SBAR tool has been supported by the creation of implementation kits in various health systems worldwide. These kits typically include training slide shows, examples of the SBAR grid, pre-designed SBAR materials for immediate use, stickers, posters, and explanatory videos. Such resources contribute to the standardized and widespread adoption of the SBAR tool by providing healthcare professionals with readily accessible materials and training support. The comprehensive nature of these kits facilitates the effective integration of the SBAR communication tool into healthcare practices [24–26].

The SBAR model has been deployed in various specialties in health care institutions: obstetrics, emergency, pediatrics, neonatology, intensive care, cardiology, anaesthesia [25, 27–30].

Numerous studies, including our own, have highlighted the SBAR tool as an indispensable instrument for facilitating effective information transfer. The tool plays a crucial role in enabling clear, efficient, relevant, and concise telephone communications between healthcare workers. Moreover, it contributes to enhancing nurses’ confidence in preparing and delivering information to doctors. This positive shift can potentially lead to a flattening of the traditional doctor-nurse hierarchy, fostering improved collaboration and a better assimilation of complex situations by doctors. The use of the SBAR tool emerges as a valuable means to promote streamlined communication and teamwork within healthcare settings [14, 26, 31–34].

The findings of our study align with recent research conducted by Raurell et al. in 2021, where they explored the impact of SBAR role-play training on interprofessional teamwork and non-technical skills through a parallel randomized clinical trial. Their results indicated improvements in teamwork skills, particularly in areas such as ‘verbalize out loud,’ ‘paraphrase,’ ‘cross-monitoring,’ and ‘role clarity’ (p < 0.001, d = 0.99; p < 0.001, d = 0.77; p < 0.001, d = 0.72; p = 0.002, d = 0.66), along with enhanced patient intervention skills (p = 0.004, d = 0.66). Additionally, the intervention group reported greater confidence in performing patient assessments (p = 0.02, d = 0.56). These findings collectively underscore the positive impact of SBAR training on interprofessional teamwork and non-technical skills [35].

Another recent clinical trial emphasizes the importance of incorporating proficiency-based progression simulations into training programs. This underscores the superior effectiveness of such simulations in enhancing proficiency with the ISBAR communication tool, particularly in high-fidelity simulation settings. This research highlights the value of tailored training approaches to ensure optimal skill development in healthcare communication tools [36].In the context of our discussion, it is pertinent to reference a comprehensive review encompassing twelve studies, including 3 randomized controlled trials and 9 quasi-experimental studies. This synthesis revealed two overarching themes—communication clarity and critical thinking—within the realm of SBAR-based simulation. The findings of this review provide valuable insights into the multifaceted benefits of SBAR-based simulation, shedding light on its impact on communication clarity and the development of critical thinking skills among healthcare professionals [37]. Our study aligns with six of the twelve referenced studies, demonstrating significant positive outcomes supporting the efficacy of SBAR-based simulation in enhancing communication clarity. **However, implementing the SBAR tool in a developing country like Tunisia faces multifaceted challenges, especially considering that all dimensions of the patient safety culture need improvement among Tunisian establishment’s professionals** [38]. **Limited resources, diverse healthcare workforce training needs, and cultural considerations complicate the adoption process. Overcoming resistance to change, integrating the tool into existing practices, and ensuring data privacy are critical hurdles** [39]. **Sustainability, long-term stakeholder engagement, and measuring the impact on patient outcomes present additional complexities** [40]. **Successful implementation requires tailored training programs, culturally sensitive approaches, and strategies for data collection and analysis within the local context. A concerted effort to address these challenges will enable the effective integration of SBAR, fostering improved communication and patient safety in the Tunisian healthcare setting.**

Several limitations are acknowledged in our study. The training session was conducted exclusively for one service, constituting a pilot study with inherent constraints associated with a limited sample size. This restricts our ability to extrapolate broader benefits of the intervention. Additionally, our study lacks an evaluation of knowledge retention over time. To address this, we propose administering follow-up assessments at intervals of 3, 6, and/or 12 months to gauge sustained knowledge and potential changes in practice.

Future efforts should involve the expansion of training initiatives to encompass multiple departments, thereby augmenting our sample size and facilitating a more comprehensive assessment of progress. To enhance professionals’ engagement, ownership, and adaptation of the SBAR tool, provisions such as distributing informational cards and stickers are recommended. Furthermore, regular follow-up sessions should be instituted to explore and address communication challenges encountered by healthcare professionals.

A proposed avenue for further investigation involves conducting a subsequent study to evaluate the impact of the SBAR tool on patient safety. This evaluation would be measured through the incidence of Healthcare-Associated Infections (HCAIs), allowing for a comparative analysis of results before and after the implementation of the SBAR tool within the healthcare establishment.

## Conclusion

In summary, this pilot study aimed to assess the implementation of the SBAR tool in the obstetrics and gynecology department of a middle-income country. Despite the longstanding endorsement by the WHO and its recognition as a standard of care in numerous countries, our findings indicate a prevalent lack of awareness regarding the SBAR tool among the majority of professionals in the service. Nevertheless, subsequent to our training intervention, a significant proportion of participants expressed satisfaction and voiced intentions to incorporate the tool into their future practice.The favorable reception of the practice, as evidenced by positive evaluations concerning clarity, relevance of communication, and time spent on the call, underscores the potential effectiveness of this initiative. Notably, this endeavor represents the pioneering exploration of its kind in Tunisia and aligns with broader projects, including the accreditation of the University Hospital. This positions the study as a pivotal step forward in advancing healthcare communication and overall quality within the region.

## Data Availability

The datasets used and/or analysed during the current study are available from the corresponding author on reasonable request.

## Abbreviations

AEHI: Adverse Events in Health Care
FIGO: International Federation of Gynecology and Obstetrics
HCWs: Health Care Workers
HCAIs: Healthcare-Associated Infections
HAS: National Authority for Health
INEAS: the National Evaluation and Accreditation Body in the Health Sector
ISBAR: Identify, Situation, Background, Assessment, Recommendation
PACS: Services Competitiveness Support Programme
SBAR: Situation-Background-Assessment-Recommendation
WHO: World Health Organization
